# Integrin α4β1/VCAM-1 Interaction Evokes Dynamic Cell Aggregation Between Immune Cells and Human Lung Microvascular Endothelial Cells at Infectious Hemolysis

**DOI:** 10.3389/fphar.2021.653143

**Published:** 2021-04-20

**Authors:** Hai-Yan Lou, Hai-Peng Yan, Long-Gui Yang, Jiang-hua Fan, William C. Cho, Zheng-hui Xiao, Shuang-Jie Li

**Affiliations:** ^1^ Emergency Center of Hunan Children’s Hospital, Changsha, China; ^2^ Department of Pediatric Intensive Care Unit, Hunan Children’s Hospital, University of South China, Changsha, China; ^3^ Department of Clinical Oncology, Queen Elizabeth Hospital, Hong Kong SAR, China; ^4^ Department of Hepatopathy, Hunan Children’s Hospital, Changsha, China

**Keywords:** integrin α4β1, methylprednisolone, cell aggregation, red blood cells, dendritic cells, endothelial cells

## Abstract

Bacterial and viral infection is a common cause of pneumonia, respiratory failure, and even acute respiratory distress syndrome. Increasing evidence indicates that red blood cells (RBCs) may contribute to immune response and inflammation. However, the precise molecular mechanisms that link RBC and hemolysis to the development and progression of inflammatory pathologies are not entirely understood. In this study, we used bacterial endotoxin, lipopolysaccharide (LPS), to mimic an infectious hemolysis and found that RBCs dynamically regulated cell aggregation between immune cells and human lung microvascular endothelial cells (HLMVEC). When RBCs were treated with LPS, integrin α4β1 was increased and was accompanied by cytokines and chemokines release (TNF-α, IL-1β, IL-6, IL-8, IFN-γ, CXCL12, CCL5, CCL7 and CCL4). Upon α4β1 elevation, RBCs not only facilitated mature monocyte derived dendritic cell (mo-DCs) adhesion but also promoted HLMVEC aggregation. Furthermore, co-culture of the supernatant of LPS pre-treated RBCs with mo-DCs could promote naïve CD4 T cell proliferation. Notably, the filtered culture from LPS-lysed RBCs further promoted mo-DCs migration in a concentration dependent manner. From a therapeutic perspective, cyclic peptide inhibitor of integrin α4β1 combined with methylprednisolone (α4β1/Methrol) remarkably blocked RBCs aggregation to mo-DCs, HLMVEC, or mo-DCs and HLMVEC mixture. Moreover, α4β1/Methrol dramatically reduced mo-DCs migration up-regulated glucocorticoid-induced leucine zipper in mo-DCs, and ultimately reversed immune cell dysfunction induced by hemolysis. Taken together, these results indicate that integrin α4β1 on RBCs could mediate cell-cell interaction for adaptive immunity through influencing cell adhesion, migration, and T cell proliferation.

## Introduction

Red blood cells (RBCs) are emerging as important modulators of the innate and adaptive immune response (Anderson et al., 2018). Generally, RBCs are not considered as adhesive cells though they express a large number of adhesion molecules (Pretini et al., 2019). Nevertheless, in a number of pathological and disease-associated circumstances such as in sickle cell disease (SCD), malaria, polycythemia vera, hereditary spherocytosis, retinal vein occlusion and diabetes mellitus, RBCs notably alter their behaviors upon stimulated, adhere to each other and consequently to the endothelium (Dean et al., 2013; Pretini et al., 2019). Chaar et al. reported that mononuclear cells and RBCs could aggregate together through cell adhesion molecule interaction in SCD (Chaar et al., 2010). Both mature RBCs and reticulocytes were involved in these aggregations through interaction with mononuclear cells, particularly monocytes. Schӓkel and colleagues have shown *ex vivo* that an excess of RBCs, mimicking the physiological conditions in the blood, could completely prevent the phenotypical maturation of the proinflammatory subset of circulating dendritic cells (DCs) (Schäkel et al., 2006). *In vivo* and *in vitro* studies show that RBCs facilitate the engagement of circulating lymphocytes within the vascular endothelium (Munn et al., 1996; Melder et al., 2000). Additionally, vesicle-derived RBCs augment mitogen-driven T cell proliferation in peripheral blood mononuclear cell (PBMC) cultures in an antigen presenting cell- and cell contact-dependent manner (Jy et al., 2011; Danesh et al., 2014). A study has showed that packed red blood cell (PRBC) supernatant potentiates proinflammatory LPS-induced cytokine secretion from PBMCs. This response is accentuated with storage duration and partially attenuated with leukoreduction (Chaar et al., 2010).

Although the mechanisms underlying the crosstalk between RBCs and immune cells in healthy and diseased hosts remain largely unknown, more than 50 types of transmembrane proteins have been reported to involve in the transport, adhesion, and structural integrity of RBCs (Pretini et al., 2019). Integrin associated proteins on RBCs are important mediators of cell adhesion. One of the most characterized RBC adhesion molecules is integrin α4β1 (or very late antigen-4, VLA-4) on the cell surface of RBCs or reticulocytes that bind to vascular cell adhesion molecule-1 (VCAM-1), thrombospondin, and fibronectin (Gee and Platt., 1995; Kumar et al., 1996). Brittain et al. report that there is an interaction between reticulocytes and monocytes both in whole blood samples and in *in vitro* adhesion assays. Such interaction is mediated by α4β1 integrin, expressed on both cell types, via a bridge of soluble fibronectin (Brittain et al., 2008). Lu/BCAM proteins are also constitutively expressed on the endothelial cell surface and interact with α4β1 integrin on young sickle RBCs, which may contribute to the abnormal adhesion of these RBCs to resting endothelium (El Nemer et al., 2007). Furthermore, RBC markers have been found to undergo a significant change during storage, which may have detrimental immunomodulatory and hemostatic effects on the transfused RBCs (Brittain et al., 2004). RBCs are a dynamic reservoir of cytokines and chemokines in which a panel of 48 cytokines, chemokines, and growth factors in the lysate, cytosol, and conditioned media of RBCs from healthy volunteers have been identified (Karsten et al., 2018). Dean et al. also identified a panel of 32 potential biological response modifiers in the supernatants of PRBC during storage. The study found that the concentrations of ICAM-1, VCAM-1, and IL-1α varied greatly among the individual storages of PRBC (Dean et al., 2013). Recently, accumulating evidences have provided insights into the role of RBCs in the regulation of immune responses, but precise mechanisms underlying RBCs’ ability to influence immune cells remain to be defined. In this study, we aimed to investigate a possible interaction between RBCs and mo-DCs and explore how hemolysis affected cell aggregation, migration, signaling pathway activation, and cytokine release. A therapeutic opportunity for reversing immune cell dysfunctions induced by hemolysis was also explored.

## Materials and Methods

### Reagents

The antibodies to CD4 FITC and CD14 BV711 were obtained from Biolegend (San Diego, CA, United States). The antibodies to integrin β1 were obtained from BD Bioscience (San Jose, CA, United States). GILZ monoclonal antibody (CFMKG15) PE, rat IgG2a isotype control PE were from eBioscience (San Diego, CA, United States). Natalizumab was obtained from Novus Biologicals (Centennial, CO, United States). ELISA kits to TNF-α, IL-6, IL-1β, IL-8, IFN-γ were from R&D SYSTEMS (Minneapolis, MN, United States). ELISA kits to C-X-C motif chemokine 12 (CXCL12), chemokine (C-C motif) ligand 5 (CCL5), chemokine (C-C motif) ligand 7 (CCL7), and chemokine (C-C motif) ligands 4 (CCL4) were from LSBio (Seattle, WA, United States). Human CD14 cell isolation Kit was from Miltenyi Biotec Inc. (Auburn, CA, United States). EasySep Human naïve CD4 T cell isolation kit was from STEMCELL Technologies Inc. (Seattle, WA, United States). The recombinant cytokines of IL-1β, IL-6, IL-8, IFN-γ, IL-4, GMCSF, and TNF-α were obtained from Sino Biological (Wayne, PA, United States). The cyclic peptide inhibitor of integrin α4β1 (azide modified peptide: MePhe-Leu-Asp-Val-Aib-Lys) (Yu et al., 2020) control peptides (cyclic MePhe-Ala-Ala-Ala-Aib-Lys), and peptide drug conjugates (PDC), cyclic peptide inhibitor of integrin α4β1 conjugated methylprednisolone (α4β1/Methrol), and control peptide/methrol were synthesized using solid-supported chemistry by InnoPep Inc (San Diego, CA, United States). Click-iT Cell Reaction Buffer Kit, Alexa 488 alkyne, Alexa 555 conjugate of wheat germ agglutinin (WGA-555), and Alexa 488 conjugate of wheat germ agglutinin (WGA-488) were purchased from Invitrogen (San Diego, CA, United States). LPS and all other chemical reagents were obtained from Sigma (St. Louis, MO, United States).

### Sample Preparation

Peripheral blood samples were collected from healthy adult donors. Ethical approval was obtained from the Hunan Children Hospital, China (Ethical approval number: HCHLL-2021-30). All donors signed a written informed consent. Whole blood of healthy donors was withdrawn via puncture of a medial cubital vein. To prepare RBC samples, blood from two donors were combined as a sample, isolated red cells were washed twice in phosphate-buffered saline (PBS) containing 2% fetal bovine serum (FBS) with buffy coat removal, and leukocyte depletion filter (BioR, Fresenius Kabi, Bad Homburg, Germany). Subsequently, cells were resuspended in PBS, counted, and then kept on ice for 1 h. For preparation of RBCs conditioned medium samples, RBCs were exposed to different concentrations of LPS for various periods of time. To prepare large volume hemolytic samples, 5 ml of 1 × 10^9^ RBCs/ml were used in assay. After being exposed to LPS, RBCs suspensions were centrifuged at 1,500×*g* for 10 min. The supernatants were harvested and filtered, designated here as control sup (C-sup, no treatment) or LPS-treated RBCs sup (L-sup) for downstream experiments.

### Isolation of CD14 Cells and Naïve CD4 T Cells

Peripheral blood mononuclear cells were isolated with Ficoll-Pague gradient centrifugation from the donors’ peripheral blood. CD14 cells were purified with the CD14 cell isolation kit (negative selection) following the instruction manual provided by Miltenyi Biotec Inc. The naïve CD4 T cells were purified with EasySep Human Naïve CD4^+^ T Cell Isolation Kit following the manufacturer’s protocol (STEMCELL Technologies Inc.). The purity of CD14 or CD4^+^ T cells was confirmed by flow cytometric analysis.

### Human Lung Endothelial Cells (HMVEC-L) Cultivation

Primary human lung microvascular endothelial cells were purchased from Lonza (HMVEC-L, CC-2527, Basel, Switzerland). The HMVEC-L cells were maintained in endothelial basal medium-2 (EBM-2, CC-3156) supplemented with endothelial growth medium (EGM-2) MV BulletKit from Lonza Clonetics (CC-3202) at 37°C and 5% CO_2_, until achieving a 70–90% confluence. The cells were then treated with Trypsin-EDTA (Lonza) and seeded in assay plates. The cells at 3–8th passages were used for the experiments.

### *Ex vivo* Hemolysis Assay

*Ex vivo* hemolysis assay was performed essentially as described by Karsten et al. (Brittain et al., 2004). Human RBCs were washed twice in PBS and resuspended in RPMI-1640 medium (Sigma) at a density of 1 × 10^9^ RBCs/mL at 4°C. One hundred microlitre of RBCs exposed to LPS (0, 5, and 50 μg/ml) in RPMI-1640 medium supplemented with 1% penicillin-streptomycin (HyClone Laboratories, Logan, UT, United States) and incubated at 37°C for 24–48 h. The supernatants were collected or stored at −80°C until measurements for free hemoglobin protein (HGP) concentration, plasma activity of lactate dehydrogenase (LDH), and the levels of cytokines and chemokines. Hemolytic activity was assessed using levels of HGP by blood cell analyzer (Sysmex 800i, Japan) and LDH by automatic biochemical analyzer (Beckmancounter AU5800, United States) at the Laboratory of Hunan Children Hospital Center, China.

### Generation of Mature Monocyte Derived Dendritic Cells

6 × 10⁶ isolated CD14^+^ monocytes were cultured in 3 ml/well of 6-well plate in complete medium (RPMI 1640, 2 mM L-glutamine, 1% autologous plasma, 1% penicillin-streptomycin) supplemented with 250 IU/ml IL-4 and 800 IU/ml GM-CSF. Cells were incubated at 37°C and 5% CO_2_ for 2 days. 1.5 ml/well of cells were resuspended in complete medium with the 2-fold concentration of IL-4 and GM-CSF and then put back to the original culture. Cells were incubated at 37°C and 5% CO_2_ for 3 days. At day 6, cells were resuspended in 1.5 ml (per well) medium supplemented with 2,000 IU/ml IL-6, 400 IU/ml IL-1β, 2,000 IU/ml TNF-α, and 2 μg/ml PGE_2_ and placed the supplemented resuspension back into the original culture and incubated for another 3 days. The phenotypes of mo-DCs were assessed by flow cytometry on day 10. The morphology of mo-DCs was defined by a light microscope image (Keyence microscope BZ-X800).

### Cell-Cell Aggregation Assay in Suspension

We recently reported a single cell suspension self-aggregation assay (Yu et al., 2020). In this study, the protocol was modified to test multiple cell suspensions for aggregation in 96-well plate. Briefly, different dyes of Hoechst, WGA-555, and WGA-488 were used to label the different live cells. Each cell suspension was immediately mixed into wells of 96-well plate with a total volume of 100 µl per well. Cell densities, medium volumes, incubation times, and treatment schedules were optimized for different experiments. Cell-cell suspensions in plates were placed on a microplate shaker to shake orbitally for 30–60 min followed by 10 s at 500 rpm to ensure that the cell aggregates and existing single cells within the wells were processed uniformly. This procedure also eliminated the non-specific cell clusters that would mechanically be dissociated into single cells while cell-cell aggregates remained in wells. Afterward, the plates were left on the bench for 20 min and subjected to imaging captures by fluorescence microscopy (Keyence BZ-X800) or cells were fixed by addition of 50 µl 3× prefer fixative for further image capture (Anatech Ltd. sop 410, Battler Creek, MI, United States). Values calculated by ImageJ (National Institutes of Health (NIH), Bethesda, MD, United States) represented the mean ± standard deviation (SD) of at least six independent experiments.

### Monocyte Derived Dendritic Cells Migration Assay

Monocyte derived dendritic cell migration assay was performed in a Boyden chamber system (Millipore QCM 24-well, Temecula, CA, United States). mo-DCs were suspended in assay medium (serum-free RPMI 1640 containing 0.05% BSA). 800 μl of indicated sample was placed in the lower chamber. To prepare the testing samples in the lower chamber, RBC supernatants were filtered using 0.45 µm filter (Millipore) to remove RBCs after being pre-treated with LPS (50 μg/ml) for 48 h in culture (L-sup) or medium as control supernatant (C-sup). 400 µl of each supernatant was diluted in 800 µl assay medium in well of 24 well plate in the lower chamber. 200 µl of 2 × 10^5^ mo-DCs labeled with 0.1 μg/ml of Hoechst in assay medium was added into the upper chamber and then incubated for 6 h. Afterwards, the plate was put in a microplate shaker to shake orbitally for 10 s at 500 rpm to ensure that the cells in wells were processed uniformly. 200 μl of cells from the low chamber well was transferred to 96 well plate and shaken for 10 s at 500 rpm and then Hoechst positive cells were counted under a Keyence fluorescence microscope (BZ-X800 Series, Itasca, IL, United States). Values calculated by ImageJ represented the mean ± SD of at least three independent experiments.

### RBCs Integrin β1 Detection

One millilitre of 1 × 10^9^/ml RBCs were exposed to LPS or PBS for 48 h. Cells were washed once using PBS and resuspended in PBS. Cells were stained with FITC anti-human β1 antibody and FITC mouse IgG1 isotype control for 60 min on ice. Cells were washed twice with 100 µl of PBS and then resuspended in 200 µl PBS for flow cytometric analysis. Values calculated by mean fluorescence intensity (MFI) represented the mean ± SD of at least three independent experiments.

### ELISA Assay

Levels of cytokines (TNF-α, IL-6, IL-1β, IL-8, IFN-γ) in RBCs supernatants were quantified using a commercial ELISA Kit according to the manufacturer’s guidelines (R&D system, Minneapolis, MN, United States). Chemokines (CXCL12, CCL5, CCL7, and CCL4 were quantified in RBCs supernatants using commercial ELISA Kit following the manufacturer’s recommendations (LSBio, Seattle, WA, United States). Human VCAM-1 was quantified in RBCs supernatants using commercial ELISA Kit following the manufacturer’s recommendations (LSBio, Seattle, WA, United States). Red blood cells were kept at a density of 5 × 10^8^ cells per well in 96-well plate in a total volume of 200 µl PBS in the presence of LPS (0, 50 μg/ml) in 37°C, 5% CO_2_ incubator for 48 h. The supernatants were harvested after centrifugation at 1,000 × g for 15 min and stored at −80°C until use for the cytokines ELISA assay. To determine cytokine concentration, each sample was diluted in PBS at different ratios for optimization. Concentration of each cytokine or chemokine was determined from a linear regression standard curve using the standard protein controls provided in the kit. Data represented the mean ± SD of three independent experiments.

### T Cell Priming Capacity in Mixed Lymphocyte Reaction (MLR) Assay

To prepare RBCs/mo-DCs culture, 1 ml of 1 × 10^9^ RBCs were exposed to LPS (0, 50 μg/ml) in well of 24-well plate in PBS at 37°C and 5% CO_2_ for 48 h. Afterwards, 50 µl of RBCs suspension was added into well of 96-well plate containing 2 × 10^4^ mo-DCs in 100 µl complete medium and co-cultured at 37°C and 5% CO_2_ overnight. The next day, 5 × 10^6^ purified naive CD4^+^ T cells were suspended in 400 µl PBS, and labeled with 100 µL of a 10 µM CellTrace Violet solution (Life Technologies) for 5 min at room temperature (RT). Subsequently, the cells were washed three times with 2 ml MLR medium (RPMI 1640, 2 mM L-glutamine, nonessential amino acids, 0.1 mM sodium pyruvate, 5% human AB serum). Finally, the CD4^+^ T cells were suspended in MLR medium at a density of 5 × 10^5^ cells/ml. Subsequently, the labeled T cells (100 µl per well) were added and cultured for 5 days at 37°C, 5% CO_2_. Proliferation of CD4^+^ T cells was determined by measuring the fluorescence of CellTrace Violet by flow cytometry. Cell debris and dead cells were excluded from the analysis by scatter signals and propidium iodide (PI) fluorescence.

### Luciferase Reporter Assay

CD4 naïve T cells were seeded in six-well plates at 2 × 10^6^ cells per well and then treated with medium only, RBCs supernatant only, mo-DCs supernatant only, LPS only, and LPS pre-treated RBCs/mo-DCs supernatant, respectively. The pretreated CD4 naïve T cells were transfected for 48 h with NF-κB luciferase reporter vector and a negative control vector provided from the NF-κB reporter kit (BPS Bioscience, San Diego, CA, United States). Cells were harvested, lysed, and prepared before subjected to the dual luciferase reporter assay (Promega, Madison, WI, United States). Luciferase activity was measured using a luminometer (Tecan, Mannedorf, Switzerland) and relative luciferase activity was calculated as the fold change over the unstimulated vehicle control.

### Intercellular GILZ Detection

One millilitre of 1 × 10^9^ RBCs was exposed to LPS (0, 50 μg/ml) in well of 24-well plate in PBS at 37°C and 5% CO_2_ for 48 h. Afterward, 50 µl of RBCs were added into well of 96-well plate containing 2 × 10^4^ mo-DCs in 100 µl complete medium and co-cultured at 37°C and 5% CO_2_ overnight. Subsequently, the α4β1/Methrol (10 µM) and controls (100 µl per well) were added and incubated on ice for 60 min, then washed with complete medium and cultured overnight at 37°C, 5% CO_2_. Afterwards, non-nucleated erythrocytes were lysed with 1-step Fix/Lyse solution at 37°C for 20 min while preserving leukocyte population. Cells were washed twice with 100 μl of Intracellular Staining Perm Wash Buffer (Biolegend), centrifuged at 350 × g for 5 min, permeabilized by exposure to 100% methanol for 90 min on ice, and washed twice with PBS. Samples were stained with anti-GILZ PE (1:200) at RT for 60 min. Samples were washed with PBS and 100 μL PBS added to each well for flow cytometric analysis.

### Generation of α4β1/Methrol

We previously reported that the cyclic peptide inhibitor, peptide C (MePhe-Leu-Asp-Val-Aib-Lys) could block integrin α4β1 mediated CD4 T cell self-aggregation by HMGB1 or LPS stimulation (Yu et al., 2020). This α4β1 inhibitor binds VCAM-1 via the “Leu-Asp-Val” tripeptide (LDV) (Wayner and Kovach, 1992; Kaneda et al., 1997; Cringoli et al., 2020). The cyclic peptide inhibitor of integrin α4β1 conjugated to Methrol (α4β1/Methrol) was synthesized using solid-supported chemistry by InnoPep Inc (San Diego, CA, United States). This conjugate had been used to target integrin α4β1 for blocking RBCs mediated cell-cell aggregation in our previous study (Yu et al., 2020). In this study, we further explored whether the molecule could reverse the immune cell disorder in hemolysis.

### Statistical Analysis

All data were presented as mean ± SD. Comparison among groups was performed by Kruskal–Wallis one-way analysis of variance. Significant differences were noted at *p* < 0.05.

## Results

### Evidence of Hemolysis Induced by Bacterial Lipopolysaccharide in Red Blood Cell Suspension

**FIGURE 1:**
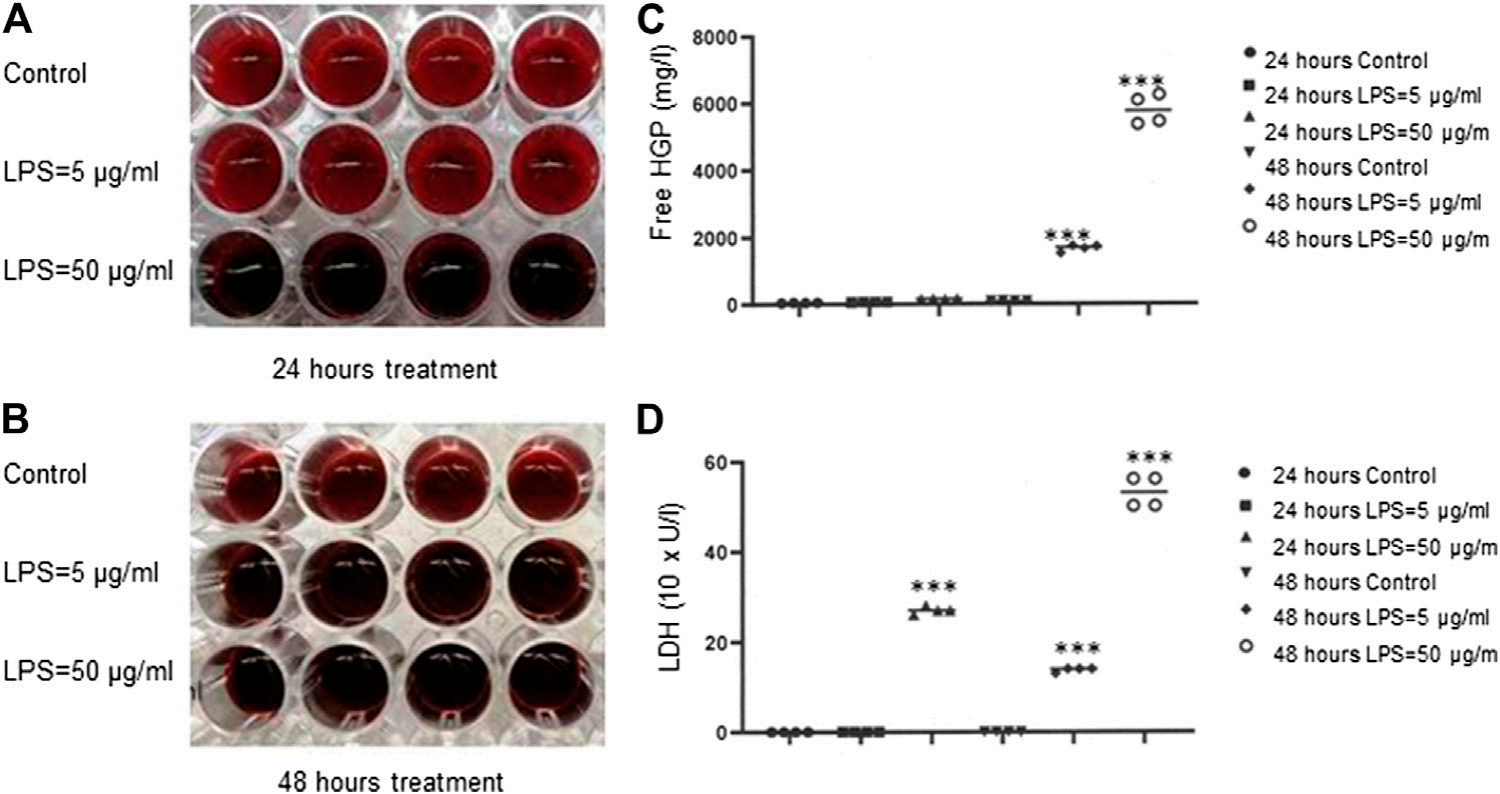
Hemolysis induced by bacteria LPS. **(A–B)** Detection of LPS-induced RBCs lysis indicated by dark red color. **(C)** Free HGP content after exposure of RBCs to LPS at 5 or 50 μg/ml for 48 h **(D)** LDH activity after exposure of RBCs to LPS at 50 μg/ml for 24 h or at 5 or 50 μg/ml for 48 h ***p < 0.001 *vs.* control. Data were representative of four blood donors.

To investigate LPS-induced hemolysis, we used red cell suspension only, which was absent of plasmatic blood components to exclude the other effectors such as complement systems and leukocytes that may contribute to hemolysis. As shown in Figure 1A,B, a dark red color was observed in the wells of RBCs treated with LPS at 50 μg/ml for 24 h, 5 μg/ml for 48 h or 50 μg/ml for 48 h compared to PBS-treated control cells. After LPS exposure, free HGP concentrations were significantly increased at both 5 and 50 μg/ml of LPS for 48 h (*p* < 0.001) (Figure 1C), and free LDH activities were dramatically increased for all the three treatment schedules (*p* < 0.001) (Figure 1D). These data provided the clear evidence that LPS evoked hemolysis *in vitro*. Our data were consistent with a previous study (Brauckmann et al., 2016).

### Integrin α4β1 on Red Blood Cells in Hemolysis Facilitates Monocyte Derived Dendritic Cells and Human Lung Microvascular Endothelial Cells Aggregation

**FIGURE 2:**
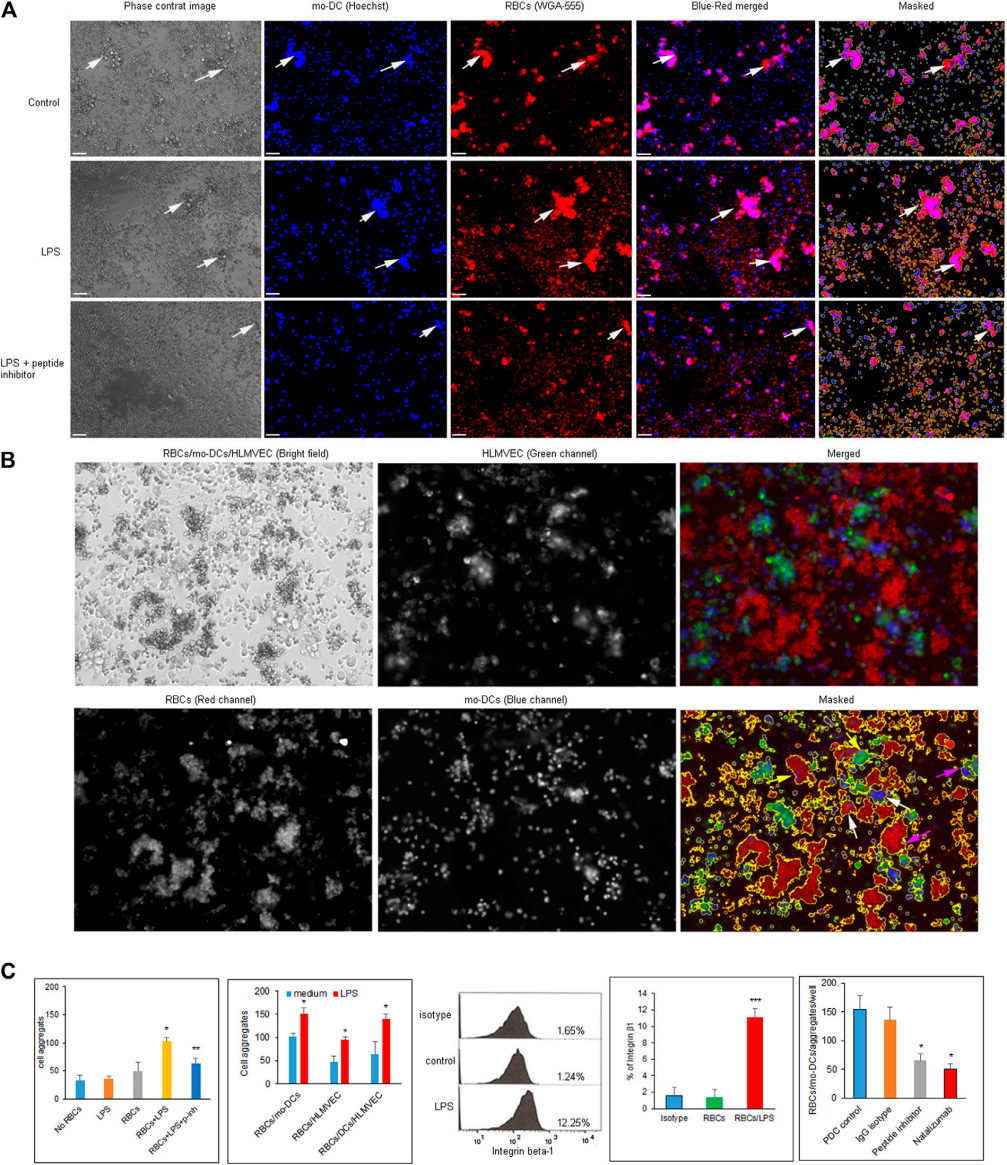
**(A)** Images of RBCs/mo-DCs aggregates captured by Keyence fluorescence microscope. RBCs and mo-DCs were pre-labeled with WGA-555 and Hoechst dye, respectively. Top panels show medium control; middle panels show LPS treated RBCs group (LPS 50 μg/ml for 24 h); bottom panels show the RBCs/mo-DCs group with RBC pretreated with 10 µM of the cyclic peptide inhibitor of integrin α4β1. White arrows pointed RBCs/mo-DCs aggregates. **(B)** The imaging strategy to distinguish cell aggregation with the corresponding three cell types in cell suspension. RBCs, mo-DCs, and HLMVEC were pre-labeled with WGA-555, Hoechst dye and WGA-488 respectively. Accordingly, aggregates were defined as RBCs/mo-DCs (white arrows), RBCs/HLMEC (yellow arrows), and RBCs/mo-DCs/HLMEC (pink arrows). **(C)** Quantification of cell aggregation calculated by imaging analysis software (left two panels). Several controls were used to access the ability of cell aggregation as indicated. Flow cytometric analysis of the integrin β1 expression on RBCs (middle panel). Integrin β1 on RBCs treated by LPS increased significantly compared to untreated control (graph bar panel, right side of flow cytometry picture). The α4β1 peptide inhibitor (10 µM) or Natalizmab (10 μg/ml) blocked cell-cell aggregation of RBCs/mo-DCs in a concentration-dependent manner while peptide control or Methylprednisolone alone had no effect on cell aggregation (Bottom right panel). All data were representative of mean ± SD, with at least three biological replicates. *p < 0.05, ***p < 0.001.

To prepare cell suspension, mo-DCs were generated from CD14^+^ monocytes after the addition of a cytokine cocktail for 10 days. Monocyte-derived dendritic cells were identified by phenotypic maturation markers such as CD83, CD11c, HLA-DR, and CD86 (all >95% purity) using flow cytometry (Supplementary Figure S2A) and by cell morphology (Supplementary Figure S2B). The purity of CD14 and CD4 naïve T cells isolated from PBMC was greater than 85 and 95%, respectively (Supplementary Figure S2C). To evaluate cell-cell aggregation in a quantitative manner, we developed imaging protocol to visualize and analyze their morphologies by using the Keyence fluorescence microcopy assisted with ImageJ software. After pre-labelling RBCs with WGA-555 and mo-DCs nuclei with Hoechst, the clustered area was determined by ImageJ software and RBCs/mo-DCs aggregates were defined and counted when the area was greater than 350 μm^2^ (Figure 2A). RBCs mixed with mo-DCs could form RBCs/mo-DCs aggregation, whereas LPS pre-treated RBCs formed larger cell aggregates with mo-DCs, which could be reduced by Integrin α4β1 peptide inhibitor. Figure 2B shows the strategy for dynamic imaging analysis for mixture of RBCs, mo-DCs, and HLMVEC. RBCs, mo-DCs, and HLMVEC were pre-labeled with WGA-555, WGA-488, and Hoechst dye, respectively. Based on cell area and combination of each color intensity, imaged objects in wells were characterized to define the diversity of cell aggregates by Image-Pro Plus software. Based on image three color fluorescence, cell aggregate objects were gated and counted as different aggregate events (event-1: red and blue color threshold for RBCs/mo-DCs; event-2: red and blue colors threshold for RBCs/HLMVEC aggregates; and event-3: red, green, and blue colors threshold for RBCs/mo-DCs/HLMVEC aggregate). Figure 2C left two panels shows image data calculation and analysis for cell aggregation. The mo-DCs per se were able to form cell self-aggregation. Compared to mo-DCs self-aggregation baseline, LPS alone or untreated RBCs did not significantly induce mo-DCs aggregation (*p* > 0.05). To our surprise, LPS treated RBCs dramatically increased RBCs/mo-DCs cell aggregation compared to the controls (mo-DC alone, LPS + mo-DCs, and RBCs/mo-DCs) (*p* < 0.05). In addition, cyclic α4β1 peptide inhibitor significantly blocked RBCs/mo-DCs aggregation induced by LPS. To further evaluate multiple cell interaction, we identified three types of cell aggregation in suspension. To test whether LPS pre-treated RBCs could affect aggregation to mo-DCs and/or HLMVEC, a multiple-cell aggregation study was performed. As expected, LPS pre-treated RBCs facilitated aggregation of mo-DCs, HLMVEC or mo-DCs/HLMVEC as compared to RBCs controls (*p* < 0.05). In the same well, the total single cell counts dropped dramatically (data not shown). The results revealed that the LPS-induced hemolysis could significantly facilitate both mo-DCs and HLMVEC aggregation. Since integrins mediate immune cell interaction and migration and up-regulation of integrin α4β1 induced aggregation of mononuclear cells and RBCs in sickle cell disease, we sought to determine whether the integrin α4β1 on RBCs is a mediator of cell-cell aggregation in hemolytic condition. As shown in Figure 2C middle panel flow cytometry picture, % of integrin α4β1 positive RBCs was increased dramatically on LPS treated RBCs compared with the controls (0.01, Figure 2C indicated panel) by the flow cytometric analysis. Of note, natalizumab, a humanized monoclonal antibody against α4 integrin that is FDA-approved medication used to treated multiple sclerosis and Crohn’s disease (Sellner and Rommer, 2019) had an activity in blocking RBCs/mo-DCs aggregation to an extent comparable to that of the cyclic peptide inhibitor of integrin α4β1 (Figure 2C last right panel). Taken together, these results confirmed that the integrin α4β1 on RBCs in LPS-induced hemolysis facilitated cell-cell aggregation.

### Hemolysis by Lipopolysaccharide Induced Monocyte Derived Dendritic Cells Migration

**FIGURE 3:**
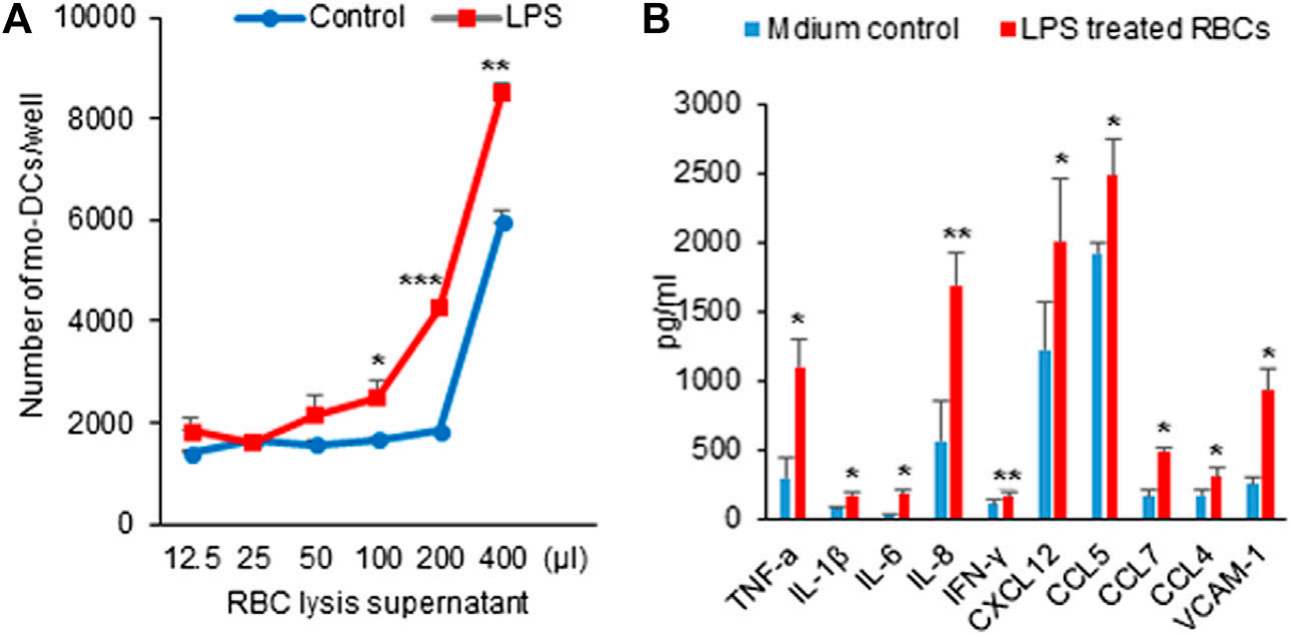
(**A**) Hemolysis by LPS induced mo-DCs migration. After overnight incubation with mo-DCs, the number of cells was counted by microscopy. L-sup induced mo-DCs migration in a concentration-dependent manner (*p < 0.05, **p < 0.01, ***p < 0.001 *vs.* the respective controls). Data were representative of mean ± SD of three experiments. **(B)** Detection of the cytokine and chemokine levels by ELISA. The cytokines (TNF-α, IL-1β, IL-6, IL-8, and INF-γ), chemokines (CXCL12, CCL5, CCL7, and CCL4), and VCAM-1 were significantly increased at L-sup (*p < 0.05, **p < 0.01 *vs.* their respective controls). Data were representative of mean ± SD from six experiments.

Chemokines are essential for mo-DCs migration. Several studies have implicated the role of DCs in progression and destabilization of the atherosclerotic plaque (Yao et al., 2011; Rai et al., 2016). A variety of factors including LPS, inflammatory cytokines, ligation of selected cell surface receptors and viral products can induce DC maturation toward a DC profile capable of inciting primary T cell responses (Yao et al., 2011). Having demonstrated that LPS could evoke hemolysis, we next sought to determine whether the hemolysis would affect mo-DCs migration. As shown in Figure 3A, the filtered L-sup induced mo-DCs migration in a concentration-dependent fashion. Figure 3B shows that a panel of cytokines that include TNF-α, IL-1β, IL-6, IL-8, IFN-r, chemokine CXCL12, CCL5, CCL7, and CCL4 were significantly increased compared to the control groups (*p < 0.05, **p < 0.01, ***p < 0.001). Therefore, RBCs lysis by LPS could release chemokines or cytokines to induce mo-DCs migration. Intriguingly, vascular cell adhesion molecule 1 (VCAM-1), known to be an endothelial ligand for integrin α4β1 that could mediate the adhesion of lymphocytes, monocytes, eosinophils, and basophils to vascular endothelium, was also increased in the filtered L-sup.

### Red Blood Cells in Hemolysis Stimulate T Cell Proliferation and Activate NF-κB Signaling Pathway

**FIGURE 4:**
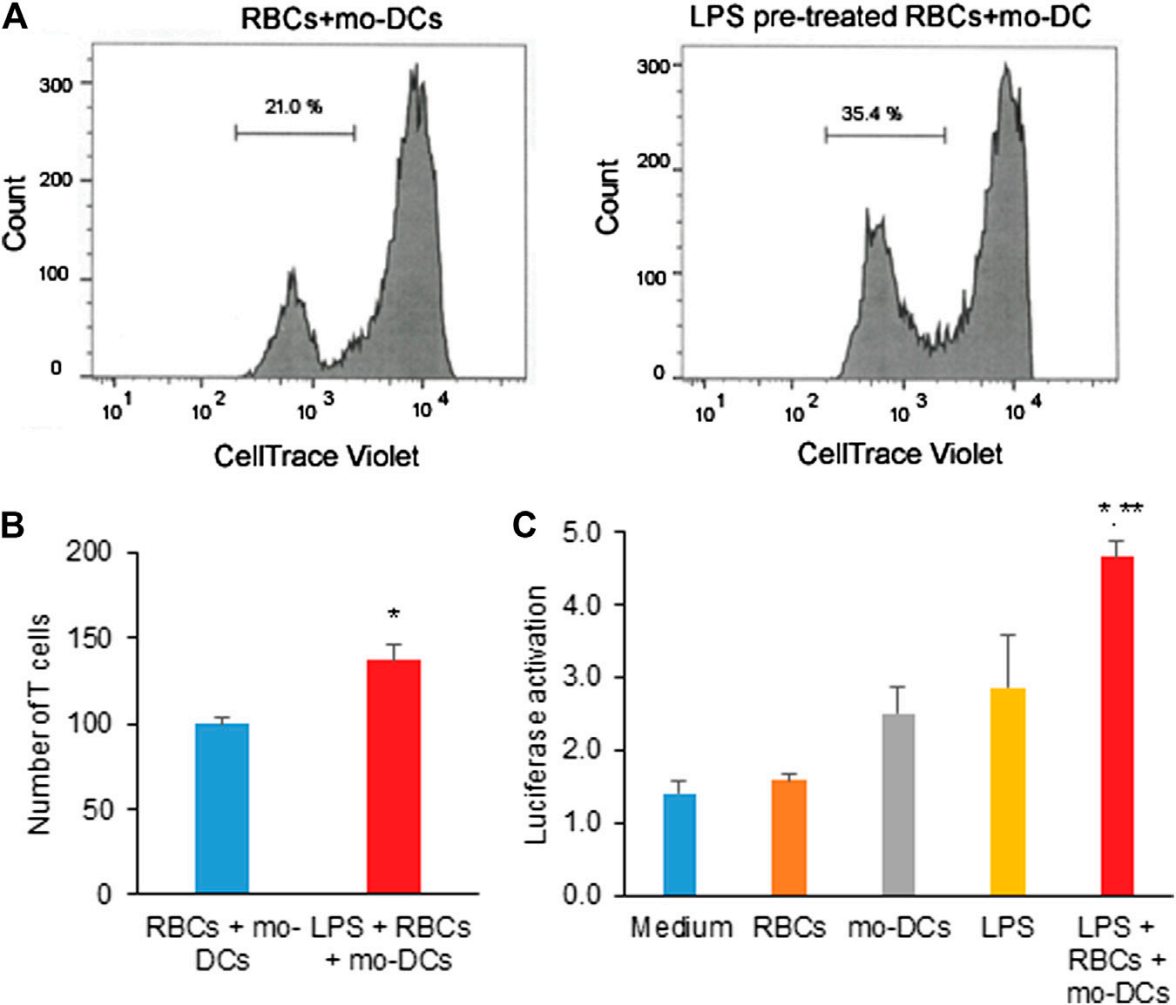
**(A)** The representative flow cytometric histograms by CellTrace Violet staining and the two violet peaks represent new **(left)** and original **(right)** generations of CD4 T cells 5 days after being grown in co-cultures of mo-DCs and LPS-pretreated or untreated RBCs. (**B**) The bar graph shows the average number of new generation of CD4 T cells determined from three independent experiments. **(C)** The effect of LPS pre-treated RBCs co-cultured with mo-DCs on the activation of NF-κB in naïve T cells by the NF-κB luciferase assay from 3 independent experiments (**p < 0.01 *vs.* controls; *p < 0.05 *vs.* LPS alone).

Given that the RBCs are the reservoirs of cytokines and chemokines, we further investigate whether RBCs in hemolysis would affect immune T cell proliferation and signaling activation. In MLR assay, naïve CD4 T cells isolated from healthy PBMC donors were co-cultured with mo-DCs in the presence of RBCs treated with LPS for five days. Figure 4A shows the representative flow cytometry data in which a new generation of CD4 T cells were stained relatively weak by Celltrace Violet. The representative result demonstrated that the culture of mo-DCs with LPS pre-treated RBCs stimulated CD4 T cell proliferation (35.56% *vs.* 21% in the untreated control group). Consistent with a previous study (Zinser et al., 2019), mo-DCs with LPS pre-treated RBCs significantly induced CD4 T cell proliferation (Figure 4B). To elucidate a mechanism responsible for the CD4 T cell activation, the NF-κB Reporter kit designed for monitoring the activity of the NF-κB signaling pathway in the cultured cells was employed. Figure 4C shows that LPS pre-treated RBCs co-cultured with mo-DCs significantly increased the NF-κB-stimulated luciferase activity (*p* < 0.01 compared to medium, RBCs alone and mo-DCs alone; *p* < 0.05 compared to LPS alone). These results suggest that RBCs in hemolysis may affect mo-DCs function and stimulate naïve T cell proliferation via activation of the NF-κB signaling pathway.

### α4β1/Methrol Reverses Monocyte Derived Dendritic Cells Dysfunctions in Hemolysis

**FIGURE 5:**
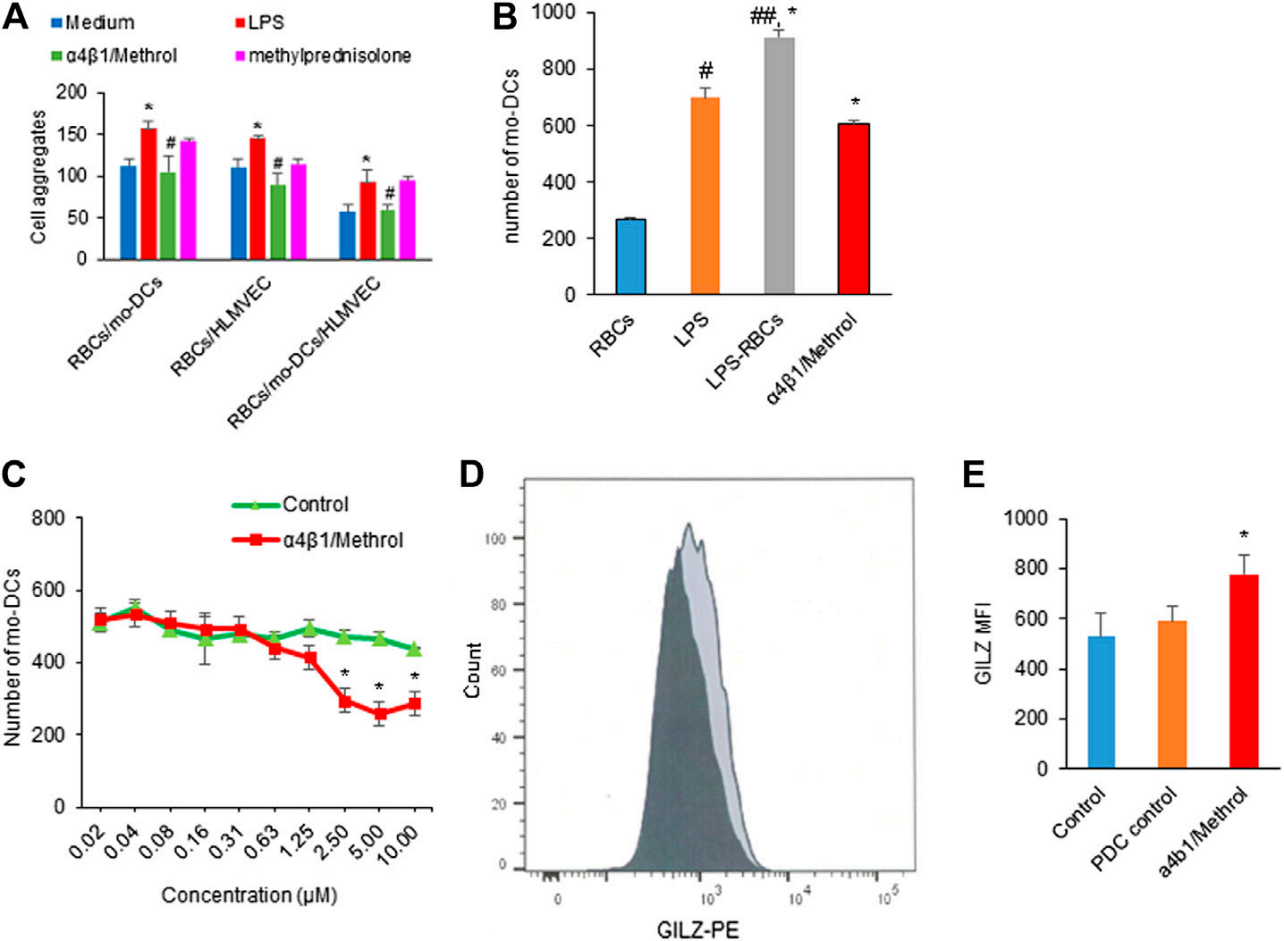
**(A)** LPS pretreated RBCs induced cell-cell aggregation on RBCs/mo-DCs, RBCs/HLMVEC, and RBCs/mo-DCS/HLMVEC (*p < 0.05 *vs.* untreated groups). Such effects were blocked by α4β1/Methrol (#*p* < 0.05). **(B)** LPS or L-sup enhanced mo-DCs migration (*p < 0.05, ##*p* < 0.01 *vs.* C-sup; *p < 0.05, L-sup *vs.* LPS alone) which was attenuated by α4β1/Methrol (*p < 0.01 *vs.* L-sup). **(C)** α4β1/Methrol decreased L-sup induced mo-DCs migration in a concentration-dependent manner [*p < 0.05 *vs.* peptide drug conjugate (PDC control)]. Data were representative of mean ± SD of three experiments. **(D)** GILZ-expressing mo-DCs analyzed by flow cytometry (dark color: PDC control; light black color: 10 µM α1β4/Methrol treatment for 24 h). **(E)** MFI values of GILZ-expressing mo-DCs quantitated by flow cytometry. α4β1/Methrol significantly increased the number of mo-DCs expressing GILZ as compared to PDC control (*p < 0.05).

Based on the observation that RBCs in hemolysis could evoke mo-DCs dysfunction, we try to formulate a strategy by using the conjugate of α4β1 integrin inhibitor and methylprednisolone to reverse the hemolysis-induced abnormal cell-cell aggregation, migration, T cell immunity and NF-κB signaling. Figure 5A shows that LPS pre-treated RBCs significantly promoted cell-cell aggregation between RBCs/mo-DCs, RBCs/HLMVEC, and RBCs/mo-DCs/HLMVEC, all of which could be blocked by α4β1/Methrol. As expected, the α4β1 inhibitor/Methrol significantly inhibited mo-DCs migration induced by the hemolytic supernatant (Figure 5B). In the mo-DCs migration assay, while LPS alone could induce mo-DCs migration the supernatant from LPS treated RBCs produced a larger effect on the cell migration (*p < 0.05). Moreover, it also dose-dependently inhibited mo-DCs migration initially promoted by L-sup (Figure 5C). Notably, the flow cytometric analysis showed that the α4β1 inhibitor/Methrol dramatically upregulated GILZ expression in the LPS pre-treated RBCs and mo-DCs cultures (Figure 5D,E, *p* < 0.05). Thus, the conjugate of cyclic peptide inhibitor of integrin α4β1 and Methrol could reverse mo-DCs dysfunctions caused by the hemolysis.

## Discussion

Currently, RBCs are emerging as an important modulator of the innate and adaptive immune response (Chaar et al., 2010; Anderson et al., 2018; Pretini et al., 2019), particularly in pathological and disease associated conditions such as PRBCs transfusion, SCD, infection sepsis, atherosclerosis, and autoimmune disorders (Anderson et al., 2018). RBCs interact with other cells leading to cell-cell aggregation and activation of aberrant signaling pathways resulting in dysfunction of immune cells (Yao et al., 2011). To investigate the interaction of RBCs and immune cells, we examined the effects of RBCs on immune cells including mo-DCs, HLMVEC, and T cells. We mimicked *in vitro* pathological conditions, i.e., LPS-induced hemolysis. After exposure of human RBCs to LPS, the evidence of hemolysis was assessed by measuring the concentrations of HGB and LDH. We found that RBCs in hemolysis increased their adhesive abilities to aggregate with mo-DC, HLMVEC, and T cells. We recently reported a method where the immune CD4 T cell self-aggregation in bloodstream induced by HMGB1 could be monitored as initial measure of immune cell activation at the early phase of inflammatory response (Yu et al., 2020). This method was able to sensitively detect immune cell self-aggregation in blood in a cell suspended status. In this study, by improving cell self-aggregation protocol, we observed cell-cell interaction under multiple cell suspension conditions. Two or even three cell types could be labeled with different dyes to track cell behaviors and to define the diversity of cell aggregates (named as different “events”). RBCs pre-treated with LPS dramatically facilitated mo-DCs or HLMVEC aggregation in cultures. To our surprise, RBCs treated with LPS promoted the mo-DCs and HLMVEC aggregation in culture compared to the untreated group. The results revealed that RBCs in hemolysis increased cell adhesive capability and promoted a crosstalk with mo-DCs and HLMVEC. The major advantage of using multiple cell aggregation imaging protocol is to provide a novel methodology for monitoring cell aggregation in blood flow when infectious hemolysis or associated inflammatory crisis occurs at an early phase of disease. Several studies have implicated the role of DCs in progression and destabilization of the atherosclerotic plaque. Dendritic cells are key sentinel cells of the innate immune system that possess the ability to stimulate the adaptive immunity. Buttari and colleagues reported that altered expression of CD47 at erythrocyte surface or its loss due to vesiculation could represent the main mechanism of the functional impairment of erythrocytes in patients via crosstalk with DCs (Buttari et al., 2015). The aggregation of RBCs to endothelial cells have been linked to various vascular disorders such as SCD, DM and hypertension. It has been recently shown that non-absorbing macromolecules can have a marked impact on mediating the aggregation efficiency of RBCs to endothelial cells in patients with type 2 diabetes mellitus (T2DM) (Pretini et al., 2019). Liu and Yang demonstrated that the dynamic interaction of RBCs and endothelial cells involves the status of glycocalyx, which could increase adhesiveness of RBCs to endothelial cells when a defect occurs (Liu and Yang, 2009). However, this has not been proven *in vivo*. It was our hypothesis that there might be an interaction between RBCs and immune cells, thus we focused the effect of integrin α4β1 on RBCs on immune cell aggregation in experimental hemolysis and found that level of integrin α4β1 on RBCs was significantly increased in hemolysis induced by LPS. Moreover, the cyclic peptide inhibitor of integrin α4β1 significantly blocked the cell-cell aggregation in RBCs/mo-DCs/HLMVEC co-cultures. The α4β1 integrin is mainly involved in the phases of leukocytes tethering and attaching on activated endothelial cells through adhesion molecules such as VCAM-1 (Harjunpää et al., 2019). Although numerous studies have reported the pathogenic role of α4β1 integrin in major inflammatory disorders (Baiula et al., 2019), very few studies have addressed the function of α4β1 integrin on RBCs. The present study revealed that α4β1 is key molecule on RBCs in LPS-induced hemolysis via triggering diverse immune response. On the other hand, VCAM-1 on RBCs exposed to LPS might also be a factor that could enhance the cell-cell interaction between RBCs and mo-DCs since mo-DCs expressed more integrin α4β1 when differentiated from monocytes.

Dynamical analysis of components of hemolysis allows us to observe the effect of RBCs on immune cells. In L-sup, the levels of cytokines TNF-α, IL-1β, IL-6, IL-8, and IFN-γ, and chemokines CXCL12, CCL5, CCL7, and CCL4 were significantly increased compared to C-sup groups. These cytokines are assumed to promote immune cell aggregation and mo-DCs migration, which might explain why the RBCs in hemolysis affect the diversity of immune response to inflammatory conditions. Under the inflammation crisis, particularly in bacterial or severe viral infection-induced hemolysis, RBCs might release excessive inflammatory mediators called a “cytokine storm” causing severe tissue damage for organ dysfunction and failure. Thus, it is crucial to monitor the level of cytokines in blood and then propose an immunomodulatory therapy to improve the outcome. It is noteworthy that an exaggerated immune response occurring in the lower respiratory tract in response to infection by coronaviruses including 2019-nCov appears to contribute to the overwhelming lung damage likely due to a cytokine storm (Girija et al., 2020). Individuals with COVID-19 have been reported to have high levels of major inflammatory cytokines such as IL-6, IL-1β, and TNF-α in the circulation (Yang et al., 2020a; Yang et al., 2020b), which was associated with the magnitude of disease severity (Wu et al., 2020). Notably, IL-6 is predominantly produced by lung epithelial cells after respiratory virus infection including SARS-CoV and MERS-CoV. In addition, it has become clear that abrupt release of IL-1β and TNF-α also contributes significantly to the severity of COVID-19 pathogenesis. In view of the current rapid global spread of SARS-CoV-2 infection and the novel coronavirus disease COVID-19 that may present with pneumonia and severe respiratory distress syndrome (ARDS), this study might be coincidentally of potential value in understanding the pathophysiology of severe COVID-19. Indeed, it has been reported that during COVID-19 infection some patients could develop autoimmune hemolytic anemia occurring within a timeframe compatible with that of the cytokine storm (Lazarian et al., 2020).

With regard to cytokine production in hemolysis, we further investigated the effect of RBCs on innate immune response. We found that the cytokines produced in culture could activate naïve CD4 T cells leading to the innate immune response. From the functional analysis of dendritic cell–T cell interaction in sarcoidosis, Kulakova et al. found that there was a markedly impaired autologous mixed lymphocyte reaction in sarcoidosis patients (Kulakova et al., 2010). DCs in a dry eye model were found sufficiently activated that stimulated the T cells involving in the onset and progression of dry eye diseases (DED) (Maruoka et al., 2018). We demonstrated that RBCs in hemolysis accompanied with cytokines release affected the function of mo-DCs to activate and promote naïve CD4 T proliferation. Dendritic cells represent the bridge between innate and adaptive immune responses. Of interest, naïve CD4 T cells were able to differentiate into T cell subsets in the RBCS/mo-DCs co-culture in response to LPS-induced hemolysis. In a preliminary experiment attempting to explore NF-κB signaling activation, we tried to test NF-κB expression in the primary T cells directly by western blot analysis using anti NF-κB antibody but unfortunately did not obtain any discernible results (data not shown). However, using the NF-κB reporter kit to measure the activity of NF-κB pathway in the CD4 naïve T cells, we found that mo-DCs in the presence of the LPS-pretreated RBCs significantly induced NF-κB activation compared to RBCs or mo-DCs alone group indicating that NF-κB signaling might play an important role in the T cell activation or differentiation.

Hemolysis can occur in a variety of pathological or disease conditions such as autoimmune hemolytic anemia, sickle cell anemia, thalassemia, glucose-6-phosphate dehydrogenase deficiency, exposure to certain chemicals, medicine, and toxins, blood clots, transfusion, and infection (Francis et al., 2013). Integrin α4β1/VCAM-1 interaction in hemolysis could evoke the immune response leading to corresponding cell dysfunction as shown in the present data and reported by others (Weingart et al., 2019; Dickmann and Bauer, 2020; Imataki et al., 2020; Yang et al., 2020). Therefore, exploration of innovative approaches to the therapy of immune disorders caused by hemolysis is highly demanded. Targeting α4β1 integrin could be a potential effective approach for treatment of infectious hemolysis since α4β1 mediates RBCs adhesion to immune cells. Komorava et al. reported that the tripeptide LDV (Leu-Asp-Val), recognized as the binding sequence found in the alternatively spliced connecting segment (CS1) region of fibronectin, is homologous and quite isosteric to the fragment IDS (Ile-Asp-Ser) present in the binding site of VCAM-1 to α4β1 (Mould et al., 1991). We recently report a modified tripeptide LDV that is bioactive small-molecule ligand to α4β1 integrin. This peptide was able to block the CD4 T cell self-aggregation induced by inflammatory stimuli in blood. Based on our observation that the cytokines/chemokines released from RBCs in hemolysis led to activation of the immune cells, we formulated a peptide-drug conjugate strategy using the cyclic peptide inhibitor of α4β1 conjugated Methrol as a potential new therapeutic modality for immune disorders, in particular infectious hemolysis. Our data shows that α4β1/Methrol molecule significantly blocked the RBCs mediated cell-cell aggregation such as RBCs/mo-DCs, RBCs/HLMVEC, and RBCs/mo-DCs/HLMVEC. Moreover, this molecule reduced mo-DCs migration remarkably. In addition, it inhibited the NF-κB activation in NF-κB transfected CD4 naïve T cells. Meyer and others reported that pulsed high-dose dexamethasone was effective in the management of chronic autoimmune hemolytic anemia of warm type (Meyer et al., 1997). Özsoylu reported that the megadose methylprednisolone for the treatment of patients with Evans syndrome (Özsoylu, 2004). However, the high dose glucocorticoids could produce GC-associated side effects.

**FIGURE 6:**
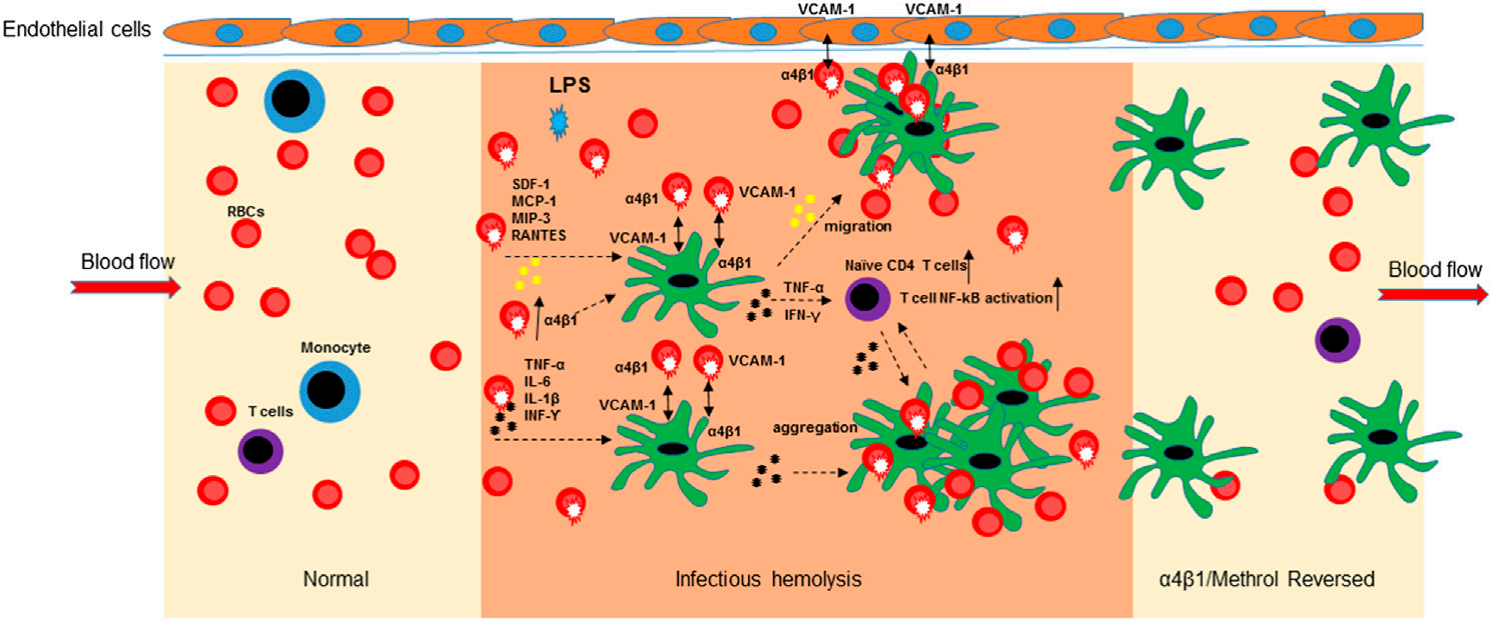
Schematic diagram of the α4β1/Methrol reversing dendritic cell dysfunction in LPS-induced hemolysis. LPS-induced hemolysis resulted in an increase of integrin α4β1 expression on RBCs that subsequently mediated cell-cell aggregation, released cytokines/chemokines and evoked immune cell responses. LPS increased integrin α4β1 expression to facilitate cell-cell interaction of RBCs/mo-DCs, RBCs/HLMVEC, and RBCs/mo-DCs/HLMVEC. Cell aggregation of RBCs and mo-DCs further enhanced cytokines release and activation of NF-kB pathway in immune T cells. Chemokines/cytokines induced mo-DCs migration to HLMVEC. α4β1/VCAM-1 is an interconnective molecule for RBCs, mo-DCs, and HLMVEC aggregation at inflamed site. α4β1/Methrol blocked cell aggregation, decreases cell migration, protected the RBCs from hemolysis caused by LPS, as well as upregulated GILZ expression culminating in reversing the dysfunction of the immune cells, in particular dendritic cells.

In conclusion, the bacterial LPS induced hemolysis is an important initial step for RBC-mediated immune response at the early phase of inflammatory responses. As pictorially modeled in Figure 6, upon overexpression of α4β1 integrin, RBCs mediate the aggregation to mo-DCs, HLMVEC and T cells and release inflammatory cytokines/chemokines, ultimately leading to mo-DCs migration, naïve CD4 T cell proliferation, and activation of NF-κB signaling.

## Data Availability

The authors acknowledge that the data presented in this study must be deposited and made publicly available in an acceptable repository, prior to publication. Frontiers cannot accept a article that does not adhere to our open data policies.

## Abbreviations

α4β1/Methrol, the conjugate of integrin α4β1 inhibitor and methylprednisolone; DC, dendritic cells; GM-CSF, granulocyte-macrophage colony-stimulating factor; HLMVEC, human lung microvascular endothelia cell; IL-6, interleukin-6; LPS, lipopolysaccharide; mo-DC, mature monocyte derived dendritic cell; PBMC, peripheral blood mononuclear cell; PRBC, packed red blood cell; RBC, red blood cell.
