# Supplementary figures and images for: Integrin α4β1/VCAM-1 Interaction Evokes Dynamic Cell Aggregation Between Immune Cells and Human Lung Microvascular Endothelial Cells at Infectious Hemolysis
Source: Front Pharmacol. 2021 Apr 20;12:653143. doi: 10.3389/fphar.2021.653143 (PMC8093802; doi:10.3389/fphar.2021.653143)

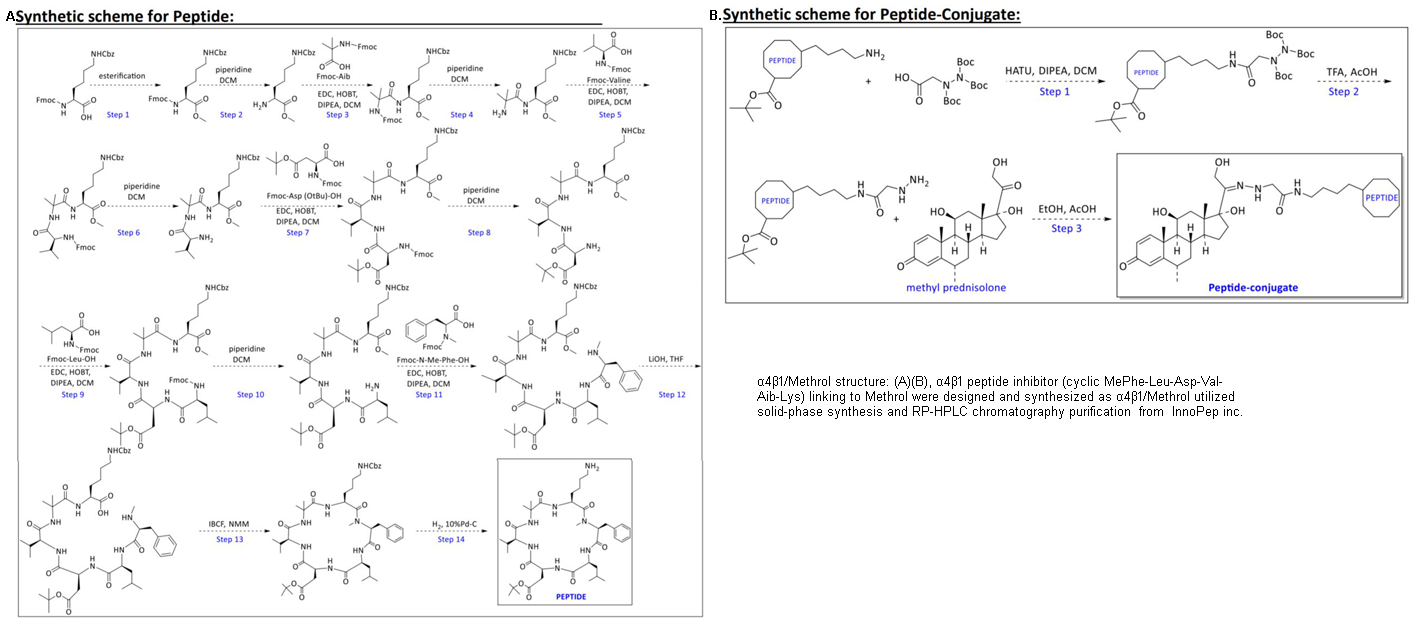

Supplement: Supplementary file 1 [file image1.tif]

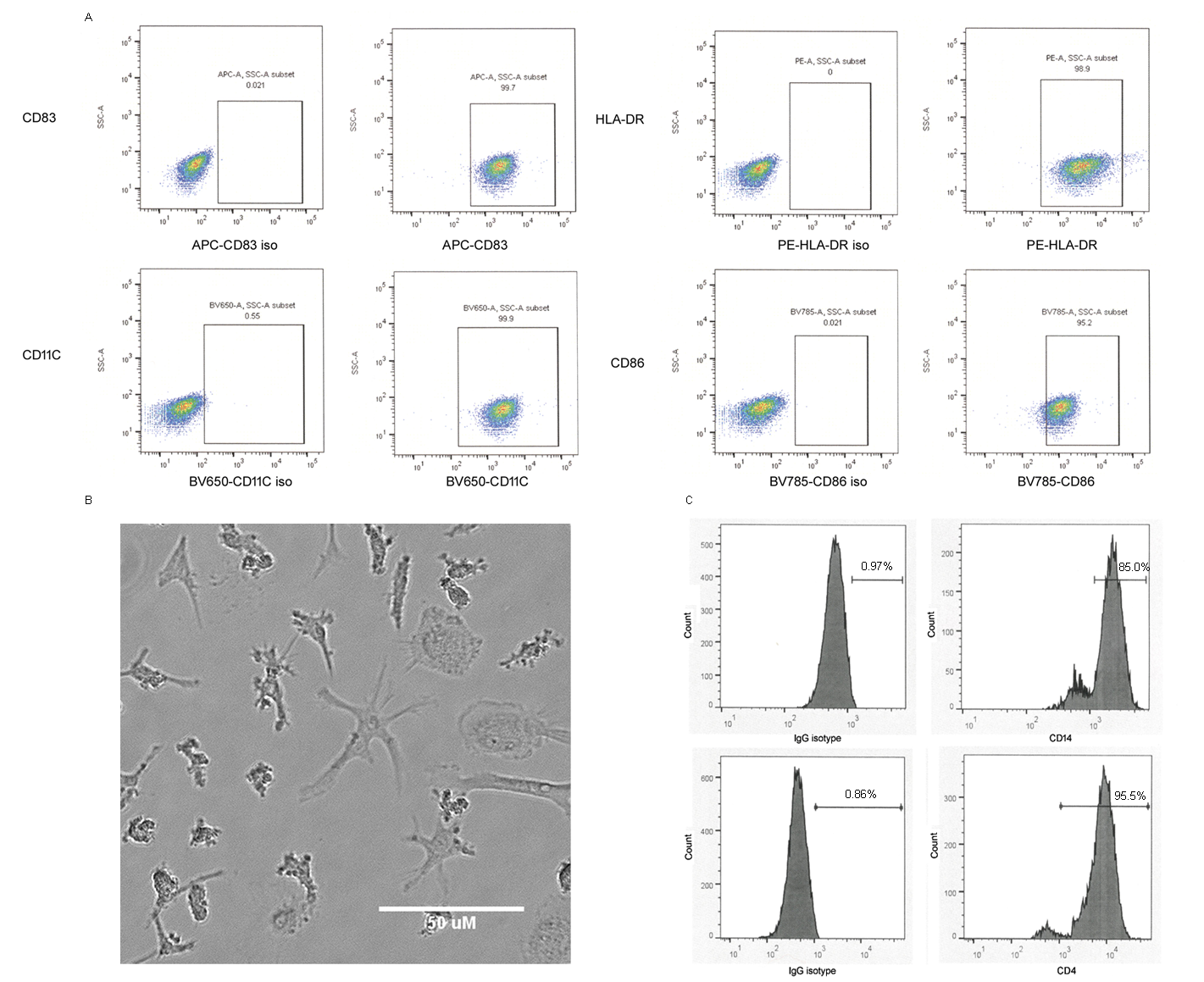

Supplement: Supplementary file 2 [file image2.tif]
